# Association of global and disease-specific health status with outcomes following continuous-flow left ventricular assist device implantation

**DOI:** 10.1186/s12872-017-0510-9

**Published:** 2017-03-14

**Authors:** Kelsey M. Flint, John A. Spertus, Fengming Tang, Philip Jones, Timothy J. Fendler, Larry A. Allen

**Affiliations:** 1 Division of Cardiology, University of Colorado Denver School of Medicine, Aurora, Colorado; Center for Cardiovascular Outcomes Research, 12631 East 17th Ave,, B130, Aurora, CO 80045 Denver, USA; 2 Saint Luke’s Mid America Heart Institute and University of Missouri - Kansas City, Kansas City, MO USA; 3 Division of Cardiology, Section of Advanced Heart Failure and Transplantation, University of Colorado Denver School of Medicine, Aurora, Colorado; Center for Cardiovascular Outcomes Research, Denver, CO USA

**Keywords:** Heart failure, Left ventricular assist device, Mechanical circulatory support, Health status, Quality of life

## Abstract

**Background:**

The prognostic value of heart failure specific and global health status before and after left ventricular assist device (LVAD) implantation in the usual care setting is not well studied.

**Methods:**

We included 3,836 continuous-flow LVAD patients in the INTERMACS registry. Health status was measured pre-operatively and 3 months post-LVAD using the Kansas City Cardiomyopathy Questionnaire (KCCQ) and EuroQol visual analog scale (VAS). Primary outcomes were mortality/rehospitalization. Inverse propensity weighting was used to minimize bias from missing data.

**Results:**

Pre-operative global and heart failure-specific health status were very poor: KCCQ median 34.6 (IQR 21.4-50.5); VAS median 43 (interquartile range (IQR) 25–65). Health status measures improved 3 months after LVAD placement: KCCQ median 69.3 (IQR 54.2-82.3); VAS median 75 (IQR 60–85). Pre-operative health status was not associated with death (unadjusted HR for lowest vs. highest score quartiles: 1.09 (0.85-1.41) KCCQ; 1.12 (0.85-1.49) VAS) or rehospitalization (unadjusted HR 0.83 (0.72-0.96) KCCQ; 0.99 (0.85-1.16) VAS). Three-month KCCQ was associated with mortality (unadjusted HR 2.17 (1.47-3.21); VAS was not (1.43 (0.94-2.17). Three-month KCCQ added incremental discriminatory value to the HeartMate II Risk Score for death (c-stat 0.60 to 0.66); VAS did not (c-stat 0.59 to 0.60). Three-month health status was associated with rehospitalization (unadjusted HR 1.31 (1.15-1.57) KCCQ; 1.24 (1.05-1.46) VAS), but did not add incremental discriminatory value (c-stat 0.52 to 0.55 and 0.54, respectively).

**Conclusions:**

These real-world data suggest that pre-operative health status has limited association with outcomes after LVAD. However, persistently low health status after surgery may independently signal higher risk for subsequent death. Further study is needed to determine the clinical utility of routinely collected health status data after LVAD implantation.

**Electronic supplementary material:**

The online version of this article (doi:10.1186/s12872-017-0510-9) contains supplementary material, which is available to authorized users.

## Background

Although survival and adverse events following left ventricular assist device (LVAD) placement have improved over time, 2-year survival after LVAD remains at 70% in the modern era [1]. Predicting mortality after LVAD is challenging, with traditional risk models achieving a c-statistic between 0.61-0.70 [2–4]. Accordingly, there are few data to guide clinicians in patient selection for this resource-intensive intervention, and current guidelines rely heavily on expert opinion [5]. Furthermore, long-term prognostication of patients who survive the acute, post-surgical phase after LVAD placement is not well-characterized. Therefore clinicians and patients often make management and goals of care decisions based upon personal, clinical and institutional experience.

One patient-reported measure that was initially hypothesized to improve prognostication following LVAD is health status. Health status encompasses symptom burden, functional status, and quality of life, and is best captured by validated questionnaires [6]. Poor health status is not only an important outcome to patients, but is also a strong independent predictor of subsequent hospitalization and mortality in patients with heart failure [7–10] and those undergoing cardiac surgery [11–14]. However, these findings do not necessarily extend to patients undergoing LVAD implantation, as pre-operative heart failure-specific health status was not associated with mortality after LVAD in the clinical trial setting [15]. Based on these findings, it has been hypothesized that heart failure treated with mechanical circulatory support may represent a relatively unique clinical situation, distinct from heart failure and other cardiac surgeries, in which the prognostic significance of health status may be largely reversed due to the profound impact of LVAD on the clinical course of the disease. However, while pre-procedure health status may not be prognostic of long-term outcomes after LVAD implantation due to a major “resetting” of patients’ health status with treatment, post-procedure health status may be associated with long-term outcomes, and assist in the planning for further treatment. Confirming these hypotheses in a contemporary, real-world setting may identify health status not only as a means for quantifying the benefits and risks of this treatment, but also as a tool to help guide clinical decision-making in the LVAD population.

To better characterize the association of pre- and post-implant health status scores with outcomes following LVAD in a usual care setting, we sought to build upon our prior work through the following: 1) confirm that, outside the clinical trial setting, pre-procedural health status was not prognostic of subsequent mortality and rehospitalization; 2) compare the relative strength of the association of global, rather than disease-specific health status with outcomes following LVAD; and 3) determine whether patients’ health status after the acute, post-surgical phase was associated with subsequent survival and rehospitalization. To address these goals, we leveraged a large, multi-center North American observational registry to examine the association between baseline and 3-month post-LVAD global and disease-specific health status measures and subsequent survival and rehospitalization.

## Methods

### Study design and population

Interagency Registry for Mechanically Assisted Circulatory Support (INTERMACS) is a prospective, quality-assurance registry of de-identified data from all patients receiving a Food and Drug Administration approved mechanical circulatory support device at 165 participating centers. Follow-up clinical data are collected after device implantation at 1 week, 1 month, 3 months, 6 months, and every 6 months thereafter. Health status data are collected pre-operatively and at 3 and 6 months after LVAD implantation and every 6 months thereafter. Hospitalization and mortality outcomes are recorded as they occur and at each follow-up period. All participating sites obtained Institutional Review Board (IRB) approval of the INTERMACS protocol. Individual patient informed consent was obtained, when mandated by the local IRB (some sites waived individual patient consent as the INTERMACS registry was viewed primarily as a quality improvement initiative). The current study did not qualify as human subjects’ research because data were de-identified and previously collected, therefore it was exempt from Colorado Multiple Institutional Review Board review. Enrollment in INTERMACS began in June 2006; however, health status reporting was not emphasized until May 2012. We therefore restricted our analysis to patients enrolled in the INTERMACS database from May 2012 to December 2013. We included all adult (≥19 years old) patients receiving a durable, continuous-flow LVAD. Baseline analyses included all patients who had baseline health status data available. Three-month analyses included only patients who survived to 3 months and had 3-month health status data available.

### Health status measures

Heart failure-specific health status was measured using the Kansas City Cardiomyopathy Questionnaire (KCCQ). The KCCQ is a self-administered questionnaire that assesses the domains of physical limitation, heart failure symptoms, social limitation, self-efficacy, and health-related quality of life. The validity, reliability, and responsiveness to change in clinical status of the KCCQ have been previously reported [16]. Answers to the questionnaire are converted into a scale of 0 to 100, with lower scores indicating worse health status. The overall summary score (OSS) was used in these analyses, and represents an average of the physical limitation, total symptoms, quality of life and social limitation domains.

The EuroQol 5-Dimensions (EQ-5D) questionnaire is a global health status measure that includes the Visual Analog Scale (VAS) [17]. The VAS asks patients to rate their overall health on a scale of 0 to 100, with 0 representing “worst imaginable health” and 100 indicating “best imaginable health.” Given the more readily interpretable data from the VAS and its proven association with mortality [18] and peak VO_2_ [19] in patients with heart failure, we used this scale as a measure of global health status.

### Statistical analysis

The distribution of KCCQ and VAS scores pre-LVAD were heavily skewed towards poor values; therefore, questionnaire answers were divided into score quartiles rather than fixed ranges. Pre-operative characteristics were compared using chi-square tests for categorical variables and one-way analysis of variance for continuous variables.

Using the Kaplan-Meier method, we measured the raw association between pre-operative and 3-month KCCQ and VAS score quartiles with mortality and rehospitalization after LVAD. Patients were censored at the time of heart transplant, device explant or recovery. We assessed the incremental prognostic value of baseline and 3-month KCCQ and VAS scores in predicting both mortality and rehospitalization when added to a base clinical model using Cox proportional hazards models. To assess whether the health status scores added prognostic information to standard clinical risk factors, we first adjusted for variables from the HeartMate II Risk Score (HMIIRS): age, albumin, creatinine, INR, and center volume [20]. We were not able to directly duplicate the HeartMate II Risk Score as the INTERMACS registry reports age as a categorical variable (50–59 years, 60–69 years, etc.) to protect patient identity, and the center volume variable in the HeartMate II Risk Score did not directly translate to the INTERMACS registry setting. Therefore, we included all of the Heartmate II Risk Score variables in a base Cox model, with age entered as a categorical variable (by decade), center volume calculated as the average number of INTERMACS-reported durable LVAD implants per year, and albumin, creatinine and INR as continuous variables.

Inverse propensity weighting (IPW) was used to decrease the effect of selection bias due to missing health status scores for both baseline and 3-month KCCQ and VAS. First, the probability of having health status scores available was calculated using a logistic regression model. Then the inverse of the probability was assigned to each patient with complete health status scores so that patients who were most like those with missing health status scores were given more weight, resulting in an IPW model that is more reflective of the general LVAD population included in the INTERMACS database. These weighted estimates were used in all analyses. IPW as a method for handling missing health status data in the INTERAMCS database has been previously validated [21] and used by other investigators [22].

Analysis was performed using SAS version 9.3 (Cary, NC). A two-sided *p*-value < 0.05 was considered significant.

## Results

### Pre-operative characteristics

The final cohort included 3,836 patients. Pre-operative global and heart failure-specific health status were very poor: VAS median 43 (interquartile range (IQR) 25–65); KCCQ median 34.6 (IQR 21.4-50.5). Health status measures improved 3 months after LVAD placement: VAS median 75 (IQR 60–85); KCCQ median 69.3 (IQR 54.2-82.3). Table 1 displays baseline patient characteristics stratified by pre-operative KCCQ score quartile. Patients in the lowest KCCQ score quartile (indicating worse health status) were more often female, INTERMACS profiles 1 or 2 at the time of device implant, and more frequently required dialysis or intra-aortic balloon pump prior to implant. Age group and implant strategy were not significantly associated with baseline KCCQ score quartile.

**Table 1:** Patient characteristics by baseline KCCQ score quartile

	Overall	KCCQ Q1	KCCQ Q2	KCCQ Q3	KCCQ Q4
N	3836	552	558	556	559
KCCQ score median (IQR)	34.6 (21.4, 50.5)*	14.3 (9.6, 18.2)	28.1 (25.3, 31.5)	41.1 (37.5, 45.6)	63.8 (56.3, 74.5)
Age in years^a^					
19-20	150 (4)	12 (2)	13 (2)	16 (3)	20 (4)
30-39	242 (6)	34 (6)	36 (7)	36 (7)	28 (5)
40-49	513 (13)	90 (16)	70 (13)	65 (12)	68 (12)
50-59	972 (25)	141 (26)	131 (24)	145 (26)	126 (23)
60-69	1330 (35)	189 (34)	207 (37)	177 (32)	212 (38)
70-79	597 (16)	80 (15)	97 (17)	111 (20)	101 (18)
≥80	32 (0.8)	6 (1)	4 (0.7)	6 (1)	4 (0.7)
Female^b^	807 (21)	148 (27)	133 (24)	97 (17)	82 (15)
INTERMACS profile^b,§^					
1 (Critical cardiogenic shock)	500 (13)	66 (12)	27 (5)	23 (4)	26 (5)
2 (Progressive decline)	1361 (36)	237 (43)	197 (34)	188 (34)	195 (35)
3 (Stable but inotrope dependent)	1193 (31)	162 (29)	201 (36)	202 (36)	213 (38)
4 (Resting symptoms)	608 (16)	73 (12)	117 (21)	110 (20)	101 (18)
5 (Exertion intolerant)	112 (3)	7 (1)	14 (3)	25 (5)	13 (2)
6 (Exertion limited)	33 (0.9)	2 (0.4)	3 (0.5)	5 (0.9)	5 (0.9)
7 (Advanced NYHA III)	29 (0.8)	5 (0.9)	5 (0.9)	3 (0.5)	6 (1)
Device strategy^a^					
Bridge to transplant	788 (21)	97 (18)	112 (20)	102 (18)	141 (25)
Possible bridge to transplant	1326 (35)	169 (31)	178 (32)	183 (33)	167 (30)
Destination therapy	1,694 (44)	282 (51)	266 (48)	269 (48)	248 (4)
Other	28 (0.7)	4 (0.7)	2 (0.4)	2 (0.4)	3 (0.6)
Dialysis within 48 h of implant^c^	40 (1)	9 (2)	1 (0.2)	1 (0.2)	2 (0.4)
Hemoglobin g/L (g/dL) ^b^	114 ± 21 (11.4 ± 2.1)	112 ± 21 (11.2 ± 2.1)	116 ± 20 (11.6 ± 2.0)	118 ± 20 (11.8 ± 2.0)	118 ± 20 (11.8 ± 2.0)
Sodium mmol/L^b^	135 ± 5	134 ± 5	135 ± 5	135 ± 4	136 ± 4
Albumin g/L (g/dL) ^b^	34 ± 7 (3.4 ± 0.7)	34 ± 06 (3.4 ± 0.6)	35 ± 7 (3.5 ± 0.7)	35 ± 6 (3.5 ± 0.6)	36 ± 6 (3.6 ± 0.6)
IABP^¶^	918 (24)	146 (26)	89 (16)	86 (16)	71 (13)
Severe depression^d^	98 (3)	23 (4)	21 (4)	12 (2)	7 (1)
Working for income^b^	572 (15)	49 (9)	72 (13)	71 (13)	109 (20)
6MWT^†^ feet^b^	802 ± 412	607 ± 517	746 ± 374	843 ± 367	908 ± 366
Gait speed m/s^e^	0.9 ± 0.8	0.6 ± 0.4	0.9 ± 1.1	0.9 ± 0.4	1.0 ± 1.0
VO_2_ max mL/kg/min^a^	11.1 ± 3.7	10.6 ± 5.2	10.6 ± 2.4	11.4 ± 4.4	11.1 ± 2.7

All data are presented as N (%) or mean ± SD unless otherwise specified. All percents are rounded to the nearest whole number

* *N* = 2225

^†^ 6 min Walk Test

^¶^ Intra-aortic balloon pump

^§^ Interagency Registry for Mechanically Assisted Circulatory Support

^a^ *p* = NS; ^b^ *p* < 0.001 ^c^ *p* = 0.005; ^d^ *p* = 0.010 ^e^ *p* = 0.008

### Missing data

At baseline, 42% of patients were missing KCCQ, and 43% of patients were missing VAS. At 3 months, 41% of patients were missing KCCQ, and 42% of patients were missing VAS. However, nearly 1/3 of the patients missing either KCCQ or VAS at 3 months did not complete any of the 3-month follow-up. Of patients who completed the 3-month follow-up, 34% and 35% of patients were missing KCCQ and VAS, respectively. Baseline KCCQ and VAS were most commonly missing due to patient and administrative reasons, whereas 3-month heath status measures were most commonly missing due to administrative reasons. The primary reasons for baseline KCCQ and VAS to be missing were: “patient too sick” (29% and 28%, respectively), and “patient consent not obtained/patient not enrolled at time QoL instrument completion due” (30% and 28%, respectively). Besides missing the entire 3-month follow-up visit, 3-month KCCQ and VAS were most commonly missing because “coordinator was too busy or forgot to administer QoL instrument” (24% and 21%, respectively), “other” administrative reasons (13% and 9%, respectively) and “reason not given” (0.7% and 25%, respectively). Patients missing baseline KCCQ data were more likely to be INTERMACS profile 1 at the time of implant, require IABP, mechanical ventilation and dialysis prior to implant and to receive LVAD for destination therapy (Additional file 1: Table S1).

### Pre-operative health status scores and mortality and rehospitalization

**Fig. 1:**
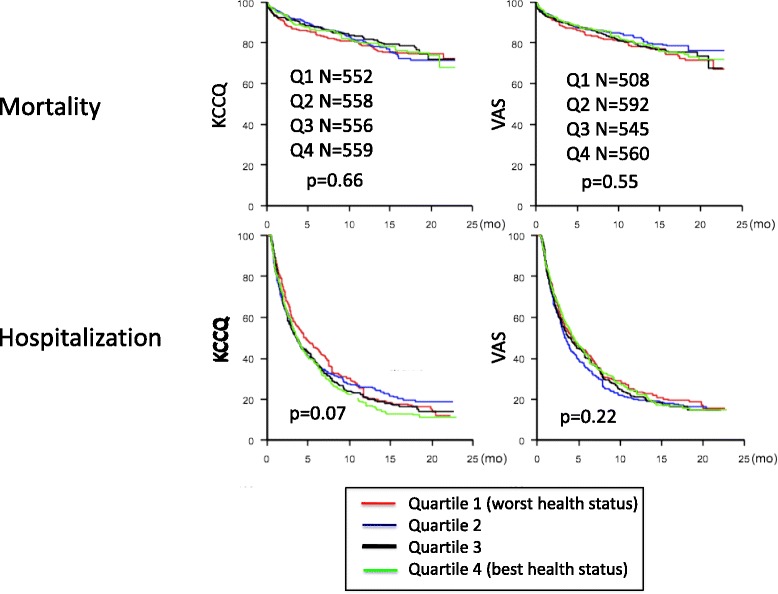
Unadjusted inverse propensity weighted Kaplan-Meier curves showing association between pre-operative (baseline) KCCQ and VAS with mortality and hospitalization

In unadjusted, IPW Kaplan-Meier analyses, pre-operative KCCQ and VAS scores were not associated with mortality or rehospitalization (Fig. 1). For mortality, the unadjusted HR for lowest vs. highest score quartile of KCCQ was 1.09 (95% CI 0.84-1.41), and was 1.12 (0.85-1.49) for VAS. For rehospitalization, the unadjusted HR for lowest vs. highest score quartile of the KCCQ was 0.83 (95% CI 0.72-0.96), and was 0.99 (0.85-1.16) for VAS. Results were similar after adjusting for the variables in the HMIIRS (data not shown). Pre-operative KCCQ and VAS did not add incremental prognostic value when added to the variables included in the HMIIRS (Table 2).

**Table 2:** Incremental prognostic value of weighted health status over the HeartMate II Risk Score in predicting mortality and rehospitalization after LVAD implantation

	C-statistic (base clinical score only)	C-statistic (base clinical + health status measure)	C-statistic (base clinical score only)	C-statistic (base clinical + health status measure)
	*Outcome:* *Mortality*		*Outcome:* *Rehospitalization*	
Baseline KCCQ (*N* = 2225)	0.60	0.61	0.51	0.50
Baseline EQ-5D VAS (*N* = 2205)	0.60	0.60	0.51	0.52
3-month KCCQ (*N* = 2060)	0.60	0.66	0.52	0.55
3-month EQ-5D VAS (*N* = 2005)	0.59	0.60	0.52	0.54

Base clinical score was comprised of the variables included in the HeartMate II Risk Score [20] – age, albumin, creatinine, center volume, INR

### Three-month health status scores and mortality and rehospitalization

**Fig. 2:**
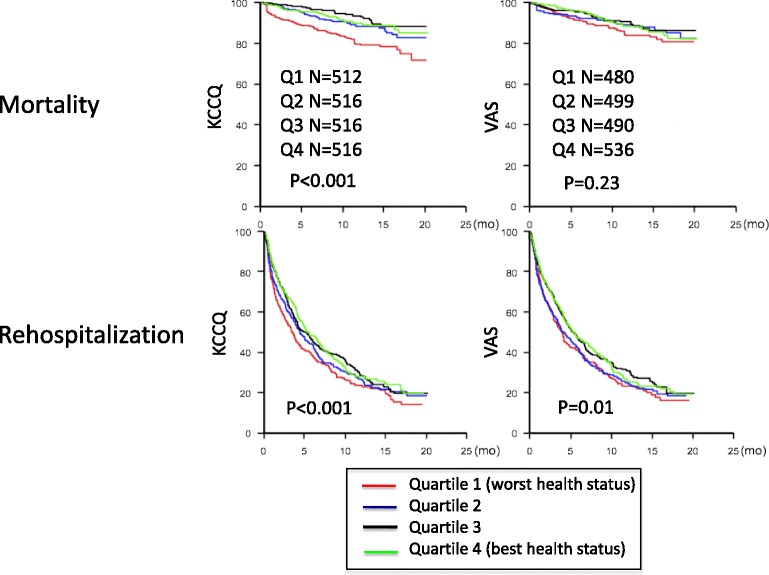
Unadjusted inverse propensity weighted Kaplan-Meier curves showing association between 3-month KCCQ and VAS with mortality and hospitalization

In unadjusted, IPW Kaplan-Meier analysis, 3-month KCCQ scores were significantly associated with mortality, which was primarily driven by the lowest health status quartile (Q1; Fig. 2). Unadjusted HR (95% CI) for the lowest vs. highest KCCQ score quartile was 2.17 (1.47-3.21), and was 2.23 (1.49-3.34) after adjusting for the variables in the HMIIRS. Three-month VAS scores, however, were not associated with mortality (Fig. 2; Unadjusted HR (95% CI) for lowest vs. highest score quartile was 1.43 (0.94-2.17)).

Three-month KCCQ and VAS were both statistically associated with rehospitalization following LVAD implantation (Fig. 2). The unadjusted HR (95% CI) for lowest vs. highest score quartile of KCCQ was 1.34 (1.15-1.57), and was 1.35 (1.14-1.60) after adjusting for the variables in the HMIIRS. For VAS, the unadjusted HR (95% CI) was 1.24 (1.05-1.46), and was 1.20 (1.01-1.43) after adjusting for the variables in the HMIIRS.

The 3-month KCCQ added incremental discriminatory value for the outcome of mortality when added to the variables included in the HMIIRS (Table 2); VAS did not. Three-month KCCQ and VAS scores did not add meaningful prognostic value for the outcome of rehospitalization (Table 2).

## Discussion

In a large, prospective registry of patients receiving a durable, continuous-flow LVAD in the usual care setting, pre-operative health status was not associated with long-term mortality or rehospitalization, and did not add incremental discriminatory value to an existing validated risk model. However, 3-month post-implant heart failure-specific health status was associated with long-term mortality, but less so with hospitalization. These real-world results confirm our prior work in the clinical trial setting, which also found no association between baseline KCCQ and mortality following HeartMate II implantation [15]. Taken together, these data suggest that very poor pre-operative health status should not, in and of itself, preclude LVAD implantation. Mechanical circulatory support may represent a relatively unique clinical situation, distinct from heart failure and other cardiac surgeries, in which the intervention is so significant that it renders pre-operative heart failure-specific health status irrelevant. The current study also suggests that serial measurements of heart failure-specific health status following LVAD implantation may help inform clinical decision-making; however, further work is needed to identify the role health status might play in guiding patients’ care once they have survived the acute, post-operative period.

### Current mortality risk prediction in the LVAD population

The HeartMate II Risk Score is one of the most commonly used risk tools for pre-LVAD assessment. It was derived from a cohort of clinical trial patients who received continuous-flow LVAD [20, 23], and has been validated in additional populations showing variable performance [3, 4, 17, 24]. When applied to 201 continuous-flow LVAD recipients at Columbia University Medical Center, the HeartMate II Risk Score had a c-statistic of only 0.56 for 90-day mortality [3], but when applied to 269 patients treated at Barnes-Jewish Hospital, the c-statistic was 0.70 for 90-day mortality, likely reflecting substantial differences in patient selection [4]. Loghmanpour et al. applied the HeartMate II Risk Score to all patients in the INTERMACS database, and reported an overall c-statistic of 0.57 (90-day mortality) and 0.60 (1-year mortality) [24], which is comparable to the results of the current study. The authors then utilized the INTERMACS database to demonstrate the significant promise of Bayesian analysis in predicting mortality in patients undergoing LVAD placement; however, these methods requires further adaptation before being easily applied in the clinical setting [24]. Given these limitations, additional efforts are needed improve prognostication in the LVAD population.

### Mortality

The current study validated results from prior work which demonstrated that pre-operative KCCQ scores did not predict mortality or rehospitalization [15]. A potential reason for this finding is that LVAD therapy often improves patients’ heart failure so significantly that pre-operative health status is no longer relevant. Moreover, the long-term outcomes of LVAD patients are often dictated by factors unrelated to the left ventricle (e.g. stroke, right heart failure, infection, bleeding, renal dysfunction) [25]; indeed adverse events due to problems with the LVAD itself are unlikely to be predicted by a pre-operative patient-reported outcome such as health status. Pre-operative health status may still aid in patient selection for LVAD because those with much better health status prior to LVAD will have less opportunity to improve, and may not derive as much health status benefit from treatment compared to those with poorer pre-operative health status [26]. On the other hand, patients with poor pre-operative health status are more likely to suffer death or low health status following LVAD [22].

Interestingly, 3-month KCCQ was predictive of mortality and added incremental prognostic value to the base clinical model. Although overall health status improves dramatically following LVAD [27], not all patients will follow such a positive trajectory. Three-month KCCQ may thus help identify patients unlikely to enjoy the expected long-term benefit from their device due to ongoing heart failure or complications of the therapy. Prospective studies involving the serial measurement of health status after LVAD may help inform decisions regarding interventions targeting health status, and guide the timing of alternative treatment options, such as transplant for eligible patients, or eventual transition to palliative care.

### Rehospitalization

In the current study, neither the base risk model nor health status measures provided meaningful discrimination for rehospitalizations in patients with a continuous-flow, durable LVAD. In general, hospitalizations for heart failure are more challenging to predict than mortality [28–32]. These problems may be magnified in the LVAD population, as the LVAD itself introduces many new reasons for why patients may be rehospitalized. Existing data report remarkably high hospital readmission rates after LVAD [33, 34]. The leading causes for hospital readmission in this population range from non-cardiac comorbidities [35], gastrointestinal bleeding [36], and device component infection [37] to recurrent heart failure [38] and progression of cardiac pathology [37]. The current study represents the challenges inherent in attempting to predict a very common outcome, and the need for further study.

### Future directions

Determining the full clinical utility of serial health status measurements beyond 3 months after LVAD will require further investigation. Ultimately, a novel heath status tool tailored to the pre- and post-LVAD populations, which incorporates broader concepts of non-heart failure aspects of frailty,[39] may be the most promising method for incorporating health status into the care of patients both before and after LVAD implantation. The design of such a tool will require careful consideration of the heterogeneous outcomes patients with LVAD suffer, and may be most successful as a series of tools designed to predict each of the most common adverse outcomes following LVAD implantation. Preliminary data by Grady et al. describe the foundation for a novel health status tool designed for the post-LVAD period [40].

### Limitations

The current study must be interpreted with care in the setting of its limitations. First, despite being the largest source of both global and disease-specific health status data in patients with LVAD, the INTERMACS registry is missing health status data in a significant minority of patients. We tried to minimize selection bias by utilizing IPW, a previously validated method for handling missing health status data that may decrease bias by preferentially weighting patients with health status measures who look like patients with missing health status measures [21, 22]. Such techniques are only partial solutions; however, despite these missing data concerns, INTERMACS has other significant advantages that make it one of the best sources to investigate this question. Second, we did not pursue analyses examining health status scores collected beyond 3 months post-LVAD due to very high rates of missing data at time points further from implantation. Finally, we were not able to separate device-related from patient-related causes of mortality and rehospitalization; therefore, we may have underestimated the predictive value of health status for patient-related adverse outcomes.

## Conclusion

The current study builds upon prior work, confirming the absence of an association between baseline health status and mortality and hospitalization following LVAD implantation, perhaps because LVAD is such a significant intervention that it renders baseline health status irrelevant to these post-LVAD outcomes. However, in patients who do survive the acute post-operative period, heart failure-specific health status does hold prognostic significance. Future studies should explore interventions aimed at identifying patients with poor health status following LVAD, and focus on further refining the use of post-operative health status measurements in the routine clinical care of this complex patient population.

## Additional file

Additional file 1: Comparison of baseline characteristics between patients with and without baseline Kansas City Cardiomyopathy Questionnaire (KCCQ) data. Baseline (or pre-operative) characteristics of patients who did and did not have baseline KCCQ data available. (DOCX 92 kb)

## Abbreviations

EQ-5D: Euroqol 5-Dimensions
HMIIRS: HeartMate II Risk Score
HR: Hazards ratio
IABP: Intra-aortic balloon pump
INTERMACS: Interagency registry for mechanically assisted circulatory support
IPW: Inverse propensity weighting
KCCQ: Kansas City cardiomyopathy questionnaire
LVAD: Left ventricular assist device
QoL: Quality of life
VAS: Visual analog scale
