# Supplementary material for: Association of global and disease-specific health status with outcomes following continuous-flow left ventricular assist device implantation
Source: BMC Cardiovasc Disord. 2017 Mar 14;17:78. doi: 10.1186/s12872-017-0510-9 (PMC5348898; doi:10.1186/s12872-017-0510-9)
Supplement: Additional file 1: — Comparison of baseline characteristics between patients with and without baseline Kansas City Cardiomyopathy Questionnaire (KCCQ) data. Baseline (or pre-operative) characteristics of patients who did and did not have baseline KCCQ data available. (DOCX 92 kb) [file 12872_2017_510_MOESM1_ESM.docx]

**Additional file 1: Table S1.** Comparison of baseline characteristics between patients with and without baseline Kansas City Cardiomyopathy Questionnaire (KCCQ) data

|  | **Baseline KCCQ** | **Missing Baseline KCCQ** | **p-value** |
| --- | --- | --- | --- |
| N | 2225 | 1611 |  |
| Age in years |  |  | <0.001 |
| 19-20 | 61 (3) | 89 (6) |  |
| 30-39 | 134 (6) | 108 (7) |  |
| 40-49 | 293 (13) | 220 (14) |  |
| 50-59 | 543 (24) | 429 (27) |  |
| 60-69 | 785 (35) | 545 (34) |  |
| 70-79 | 389 (18) | 208 (13) |  |
| ≥ 80 | 20 (0.9) | 12 (0.7) |  |
| Female | 460 (21) | 347 (22) |  |
| INTERMACS profile^§^ |  |  | <0.001 |
| 1 (Critical cardiogenic shock) | 142 (6) | 358 (22) |  |
| 2 (Progressive decline) | 811 (36) | 550 (34) |  |
| 3 (Stable but inotrope dependent) | 778 (35) | 415 (26) |  |
| 4 (Resting symptoms) | 401 (18) | 207 (13) |  |
| 5 (Exertion intolerant) | 59 (3) | 53 (3) |  |
| 6 (Exertion limited) | 15 (0.7) | 18 (1) |  |
| 7 (Advanced NYHA III) | 19 (0.9) | 10 (0.6) |  |
| Device strategy |  |  | <0.001 |
| Bridge to transplant | 452 (20) | 336 (21) |  |
| Possible bridge to transplant | 697 (31) | 584 (36) |  |
| Destination therapy | 1065 (48) | 629 (39) |  |
| Other | 9 (0.4) | 17 (1) |  |
| Dialysis within 48 hours of implant | 13 (0.6) | 27 (2) | 0.001 |
| Hemoglobin g/dL | 11.6 ± 2.1 | 11.2 ± 2.1 | <0.001 |
| Sodium mmol/L | 135 ± 5 | 135 ± 5 | 0.830 |
| Albumin g/dL | 3.5 ± 0.6 | 3.4 ± 0.7 | <0.001 |
| IABP^¶^ | 383 (17) | 526 (33) | <0.001 |
| Severe depression | 63 (3) | 35 (2) | 0.202 |
| Working for income | 301 (14) | 271 (17) | <0.001 |
| 6MWT^†^ feet | 796 ± 414 | 838 ± 393 | 0.304 |
| Gait speed m/s | 0.9 ± 0.9 | 0.8 ± 0.5 | 0.745 |
| VO_2_ max mL/kg/min | 11.0 ± 3.7 | 11.5 ± 3.6 | 0.184 |
| ^¶^Intra-aortic balloon pump  ^†^6-minute walk-test  ^§^ Interagency Registry for Mechanically Assisted Circulatory Support | | | |
